# The Effect of Thiamine Concentration on the Antioxidative Activity Indices in Tea Extracts

**DOI:** 10.3390/antiox8110555

**Published:** 2019-11-15

**Authors:** Justyna Piechocka, Krystyna Szymandera-Buszka, Joanna Kobus-Cisowska, Anna Gramza-Michałowska, Anna Jędrusek-Golińska

**Affiliations:** Department of Gastronomy Science and Functional Food, Faculty of Food Science and Nutrition, Poznań University of Life Sciences, ul. Wojska Polskiego 31, 61-624 Poznań, Poland; justyna.piechocka@up.poznan.pl (J.P.); joanna.kobus@up.poznan.pl (J.K.-C.); angramza@up.poznan.pl (A.G.-M.); anna.jedrusek-golinska@up.poznan.pl (A.J.-G.)

**Keywords:** oils, thiamine, ethanol tea extracts, antioxidants, oxidative stability

## Abstract

The aim of the study was to determine correlations between the concentration of thiamine in systems and indicators of the antioxidative activity of ethanol tea extracts in the presence of soybean oil. Variability of the thiamine form was assumed by comparison of the influence of thiamine hydrochloride or thiamine pyrophosphate and fermentation of ethanol tea extracts. The study provides practical knowledge about the antioxidative activity of ethanol tea extracts in products containing fat and thiamine. The study showed that all tea extracts exhibited higher antioxidative activity in the presence of thiamine amounts of 0.1 and 0.8 mg/100 g. The antioxidative activity of ethanol tea extracts was significantly reduced when the concentrations were higher than the natural level for foods (over 1.0 mg/100 g). The systems containing white tea extract were the most vulnerable, whereas those with black tea were the least vulnerable. The presence of thiamine pyrophosphate in the system was more strongly correlated with reduced activity of the extracts than the presence of thiamine hydrochloride.

## 1. Introduction

Teas and tea extracts are known and commonly used as sources of antioxidants [1,2,3,4]. Tea leaves at various degrees of fermentation (oxidation) have a health-promoting effect as they considerably reduce the risk of various diseases, including cardiovascular diseases and cancers [5,6,7]. Tea polyphenols capture reactive oxygen and nitrogen species, chelate metal ions and inhibit the activity of cellular kinases and pro-oxidative enzymes such as inducible nitric oxide synthase, lipoxygenase, cyclooxygenase and xanthine oxidase. They also induce phase II in enzymes and antioxidants, such as glutathione and S-transferase [8]. Tea extracts, especially ethanol extracts, are components of supplements and nutritional additives [9], which also contain other ingredients, such as unsaturated fatty acids, mineral components and vitamins. Moreover, such products often contain active forms of thiamine, which is used to treat various ailments, e.g., mild vitamin B1 deficiencies [10,11], or when it is necessary to apply parenteral hyperalimentation [12]. Very high doses of up to 3 g/day are applied to treat various diseases [11]. Thiamine exhibits some antioxidative properties, but they are rather poor, as compared with vitamin C [4]. Enthalpies and Gibbs free energies of four common antioxidant reactions, i.e., formal hydrogen transfer (FHT), proton transfer (PT), single electron transfer (SET) and radical adduct formation (RAF), have been calculated but in the aqueous phase. The results showed that in comparing with trolox and ascorbic acid, their antioxidant potential has been classified in the decreasing order: ascorbic acid > thiamine > trolox [13]. Preliminary studies indicated the existence of certain correlations between the activity of antioxidants and the content of vitamins [14,15,16]. In view of the need to apply antioxidants, including highly popular ethanol tea extracts, and their high activity, it appears important to understand the influence of various factors affecting the amount of thiamine and the antioxidative activity of these compounds. There have been numerous studies analysing the antioxidative activity of tea extracts, especially ethanol extracts [17,18,19,20], but none of them provided a detailed analysis of changes that may take place in the thiamine-tea extract systems at various levels of oxidation. The studies showed interactions between antioxidants and vitamin C [21]. The results showed that the addition of antioxidants shows impact on the thiamine reduction losses since it slows down lipid oxidation to a significant extent. Preliminary data indicate that thiamine at high concentrations slows down antioxidant activity of ethanol tea extracts [22]. There have been no reports on differences in the antioxidative effect resulting from the thiamine form, such as thiamine pyrophosphate or hydrochloride.

The authors of this study hypothesised (H0) that the concentration of thiamine and its form was correlated with the antioxidative activity indices in ethanol tea extracts at *p* < 0.05. In case the hypothesis was rejected, alternative hypotheses (H1) assumed the absence of correlations between these variables.

## 2. Materials and Methods

### 2.1. Sample Preparation 

Thiamine hydrochloride and thiamine pyrophosphate (Merck) were assumed as thiamine models. The study was conducted in model systems, where soybean oil (Flota Logistic Sp. z o.o. Tychy, Poland) systems with ethanol tea extracts (0.002%) and thiamine were adopted in weight. Tea leaves (*Camelia sinensis* L.) of different degrees of fermentation were purchased from retail outlets and used in the study. Extracts from white (China Bai Mu Dan), green (China Gunpowder Temple of Heaven), yellow (China Huang Xiao), red (China Da Hong Pao) and black (China Yunnan Gold) leaf teas were obtained using ethanol (POCH, Poland) [2], according to the procedure described by Gramza et al. [23]. The ethanol extracts were prepared by 24-h maceration of tea leaves (100 g) with 250 mL of 95% ethanol. Thiamine at different concentrations was put into soybean oil with the ethanol tea extracts. They were added at the following amounts: 0.01, 0.02, 0.04, 0.06, 0.08, 0.1, 0.2, 0.4, 0.8 (0.01–0.8 mg/100 g—natural thiamine level in food products); 1.0, 2.0, 3.0, 4.0, 6.0, 8.0, 9.0, 13.5, 16.0, 18.0, 20.0 mg/100 g (1.0–20.0 mg/100 g—enriched products). The ethanol tea extracts were added at the following amount: 0.002%.

The samples were stored at 30 ± 1 °C, without access to light. In order to obtain a uniform weight, 20 g samples with a 1.3–1.5 cm thick layer were mixed continuously. They were stored and the degree of soybean oil oxidation was measured on the following days: 0, 5, 7, 11, 15, 17, 21, 25, 28 and 31.

### 2.2. Methods

On the set days soybean oil oxidation indices were determined: the peroxide value [24] and the anisidine value [25]. The protection factor (Wo) was calculated on the basis of the lipid oxidation indicators. The antioxidative efficiency of the additives used in the model together with thiamine with oil at individual concentrations was expressed as protection factor Wo, which was the ratio between the time necessary for a particular sample to reach the peroxide value 50(meq02/kg) and the corresponding time for the control sample. Wo > 1 indicates antioxidative properties of the additive, whereas Wo < 1 indicates pro-oxidative properties of the additive [26]. The results were analysed statistically (Pearson’s linear correlation coefficients). A negative correlation value indicated that the protection factor of the ethanol tea extracts was reduced in comparison with the sample without thiamine. Apart from that, the influence of the amount of thiamine on the chelating properties [27] and reducing power [28] as well as free radicals scavenging indices (the ABTS^+^ scavenging capability [29], the DPPH scavenging capacity [30,31]) of the tea extracts were investigated. In order to compare the influence of elevated amounts of both thiamine forms on the antioxidative properties of the tea extracts, the differences between the protection coefficient of the extract and a sample without thiamine as well as a sample with thiamine added at an amount of 2.0 mg/100 g of oil were calculated. A greater difference indicated higher ‘vulnerability’ of the extract on the high doses of thiamine hydrochloride or pyrophosphate.

The results were analysed statistically with the STATISTICA^TM^ PL 12 (StatSoft) software. In order to determine the strength of the correlation between the variables, Pearson’s linear correlation coefficients (*r*) were calculated for: *r* < 0.200 no linear relationship; 0.200 ≤ *r* < 0.400 weak linear dependence; 0.400 ≤ *r* < 0.700 moderate linear dependence; 0.700 ≤ *r* < 0.900 significant linear dependence; *r* ≥ 0.900 very strong linear dependence; at: *p* ≤ 0.05; *n* = 12.

## 3. Results

The results showed the antioxidative effect of all the tea extracts. The results presented in the Table 1, Table 2, Table 3 and Table 4 relate to the samples after 30 days of storage. There was a significant correlation between the content of thiamine hydrochloride or thiamine pyrophosphate and the oxidative stability of soybean oil in the presence of the tea extracts (Table 1 and Table 2). There was no increase in the lipid oxidation indicators in the systems containing thiamine hydrochloride at the amounts corresponding to the natural level of thiamine in food products (0.01–0.8 mg/100 g).

At concentrations of 0.1 and 0.8 mg/100 g there was a decrease in the oil oxidation indices and an increase in the protection factor (Wo) in all the tea extracts. It was particularly pronounced for thiamine pyrophosphate. In the systems containing thiamine within the concentration ranges of enriched products (0.8–20.0 mg/100 g) there was a statistically significant positive correlation between the indicators of primary (the peroxide value) and secondary (the anisidine value) oxidation products of soybean oil systems with the addition of tea extracts and the content of thiamine hydrochloride and thiamine pyrophosphate. The statistical analysis revealed a negative correlation between the protection factor of the ethanol tea extracts and the content of thiamine (Table 1 and Table 2, and Tables S1–S4).

**Figure 1:**
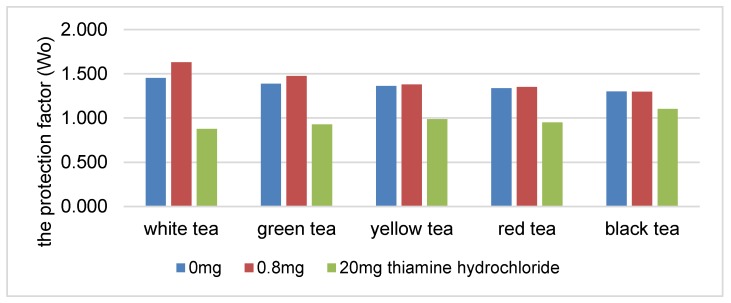
The protection factor (Wo) of ethanol tea extracts in the presence of thiamine hydrochloride.

**Figure 2:**
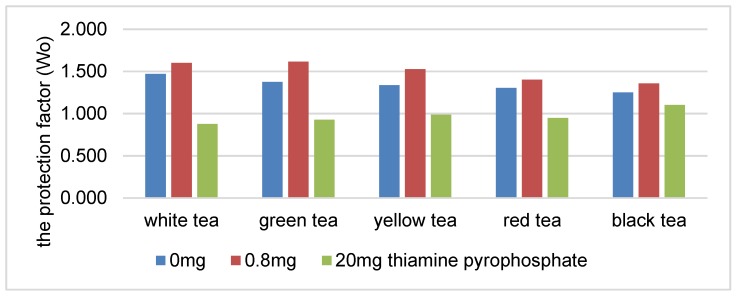
The protection factor (Wo) of ethanol tea extracts in the presence of thiamine pyrophosphate.

There was a statistically significant decrease in the protection coefficient of the tea extracts as the concentration of thiamine hydrochloride or thiamine pyrophosphate increased (Figure 1 and Figure 2). The highest correlation was found in the presence of white, green and yellow tea extracts. An increase in the content of thiamine hydrochloride from 0.8 to 20 mg/100 g reduced the protection coefficient of the white tea extract relative to soybean oil by 29% (Figure 1). The protection factor was reduced by 45% in the samples containing thiamine pyrophosphate (Figure 2).

The higher correlation coefficient in the systems containing thiamine pyrophosphate may indicate a stronger correlation between the increased content of thiamine pyrophosphate (over 1.0 mg/100 g) in the system and reduction of the antioxidative activity of the tea extracts.

Iron and copper chelation significantly influences the course of the oxidation process and the compounds binding metals are classified as inhibitors of the oxidation process [32]. These compounds can be found in polyphenols contained in tea [2,33]. Thus, by examining the influence of high concentrations of thiamine hydrochloride and thiamine pyrophosphate, their chelating properties and reduction of the power of the extracts were taken into account (Table 3 and Tables S5–S8). Thiamine hydrochloride and thiamine pyrophosphate at the amounts of 0.01–0.06 mg/100 g did not have statistically significant effect on the chelating properties and reducing powers of the extracts.

However, thiamine pyrophosphate at the amounts of 0.08–0.8 mg/100 g caused a statistically significant increase (of approximately 9%) in the chelating properties and reduced the power of the tea extracts. There were similar but lesser (7%) tendencies in the samples containing thiamine hydrochloride. On the other hand, in the samples containing thiamine hydrochloride at the amounts of 0.8–20.0 mg/100 g the chelating properties and the power of the tea extracts decreased by 18%. There was a similar (20%) decrease in the chelating properties and power of the tea extracts in the systems containing thiamine pyrophosphate.

Free radical scavenging capability is one of the most important traits determining high antioxidative properties [34,35]. The experiment showed the antiradical effect of the tea extracts against DPPH● and ABTS●. However, analogously to the previous results, the high concentrations of thiamine hydrochloride (1.0–20.0 mg/100 g) and thiamine pyrophosphate (2.0–20.0 mg/100 g) had negative effect on the reduction activity of the extracts. These results suggest that thiamine hydrochloride and thiamine pyrophosphate at higher concentrations may exhibit significant effect on the components reducing the activity of the extracts. The higher correlation coefficient (Table 4 and Tables S9–S12) in the systems containing thiamine pyrophosphate may indicate a stronger correlation between the increased content of thiamine pyrophosphate (over 1.0 mg/100 g) in the system and reduction of the free radical scavenging capacity of the tea extracts.

The results of the study showed that high concentrations of thiamine hydrochloride and thiamine pyrophosphate in the systems significantly reduced the protection factor, chelating properties, and antiradical effect against DPPH● and ABTS● of the tea extracts. The type of the tea extracts significantly influenced the reduction of their antioxidative properties in the presence of high concentrations of both thiamine forms. The most pronounced correlation was observed in the systems containing white tea extracts. The least significant correlation was observed for the black tea extracts. A lower correlation coefficient may indicate a less pronounced correlation between increased content of thiamine hydrochloride and thiamine pyrophosphate and reduced antioxidative activity of the black tea extract. The white and green tea extracts were characterised by the greatest vulnerability, whereas the black tea extracts were the least vulnerable. These extracts exhibited higher vulnerability to thiamine pyrophosphate.

## 4. Discussion

The research results were in line with the results of earlier research on the antioxidative activity of tea extracts [18]. Moreover, the white and green tea extracts were characterised by higher antioxidative activity [36]. Earlier research showed that the efficiency of peroxide anion binding by tea extracts decreased in the following type-dependent order: oolong tea > green tea > black tea [37,38,39,40]. Toyama et al. found that a black tea extract exhibited poorer DPPH binding capability than a green tea extract [41,42,43]. The results of our research showed that the tea extract type affected the reduction of its antioxidative properties in the presence of high concentrations of both thiamine forms. The highest value of the correlation coefficient was noted in the systems containing white and green tea extracts, whereas the lowest was found in the ones containing the black tea extract. The correlations may stem from the variable composition of tea extracts resulting from the advancement of the fermentation process [14,42,43]. The content of epigallocatechin gallate decreases while the content of caffeine increases with the degree of tea fermentation [40]. The decrease in the antioxidative properties of the extracts may indicate high activity of thiamine hydrochloride and pyrophosphate against the active components of the extracts. Epigallocatechin gallate is the dominant antioxidative compound in tea extracts [44]. The highest value of the correlation coefficient in the systems containing the white tea extract may have been caused by the formation of a complex of epigallocatechin gallate and the thiamine forms. The preliminary research confirmed the possible formation of a complex of thiamine and epigallocatechin gallate [22,45]. Earlier theoretical studies showed the possible formation of hydrogen-bonded complexes of thiamine with myricetin [46].

## 5. Conclusions

The negative effect of higher than natural thiamine levels on the antioxidative activity of ethanol tea extracts should be taken into consideration.

## Figures and Tables

**Table 1:** Correlation coefficients between oxidative stability values of soybean oil and content of thiamine hydrochloride in presence of ethanol tea extracts.

		Correlation Coefficient of Oxidative Stability Values of Soybean Oil and Content of Thiamine		
Type of Tea Extracts	Concentration of Thiamine [mg/100 g]	Peroxide Value [meqO_2_/kg]	Anisidine Value	The Protection Factor (Wo)
Extract of white tea	0.08–0.8 mg	−0.929	−0.833	0.961
	0.8–20 mg	0.949	0.948	−0.908
	0–0.06 mg	−0.154	−0.123	0.155
Extract of green tea	0.08–0.8 mg	−0.674	−0.713	0.622
	0.8–20 mg	0.943	0.951	−0.913
	0–0.06 mg	−0.111	−0.077	0.111
Extract of yellow tea	0.08–0.8 mg	−0.846	−0.750	0.710
	0.8–20 mg	0.906	0.796	−0.902
	0–0.06 mg	0.032	−0.099	−0.033
Extract of red tea	0.08–0.8 mg	−0.683	−0.696	0.586
	0.8–20 mg	0.845	0.661	−0.857
	0–0.06 mg	−0.099	−0.099	0.100
Extract of black tea	0.08–0.8 mg	−0.491	0.139	0.151
	0.8–20 mg	0.646	0.613	−0.611
	0–0.06 mg	−0.207	0.069	0.207
Without additionals of extract	0.08–0.8 mg	−0.624	0.817	0.729
	0.8–20 mg	−0.260	−0.355	0.363
	0–0.06 mg	0.987	0.906	−0.975

**Table 2:** Correlation coefficients between oxidative stability values of soybean oil and content of thiamine pyrophosphate in presence of ethanol tea extracts.

		Correlation Coefficient of Oxidative Stability Values of Soybean Oil and Content of Thiamine		
Type of Tea Extracts	Concentration of Thiamine [mg/100 g]	Peroxide Value [meqO_2_/kg]	Anisidine Value	The Protection Factor (Wo)
Extract of white tea	0.08–0.8 mg	−0.900	−0.779	0.910
	0.8–20 mg	0.977	0.964	−0.954
	0–0.06 mg	0.072	−0.175	−0.073
Extract of green tea	0.08–0.8 mg	−0.974	−0.801	0.980
	0.8–20 mg	0.962	0.968	−0.916
	0–0.06 mg	−0.077	−0.077	0.069
Extract of yellow tea	0.08–0.8 mg	−0.913	−0.767	0.921
	0.8–20 mg	0.928	0.964	−0.885
	0–0.06 mg	−0.088	−0.099	0.089
Extract of red tea	0.08–0.8 mg	−0.971	−0.702	0.971
	0.8–20 mg	0.872	0.939	−0.856
	0–0.06 mg	0.013	0.070	−0.013
Extract of black tea	0.08–0.8 mg	−0.633	−0.272	0.639
	0.8–20 mg	0.764	0.832	−0.741
	0–0.06 mg	−0.271	0.180	0.271
Without additionals of extract	0.08–0.8 mg	−0.403	−0.107	0.384
	0.8–20 mg	0.948	0.938	−0.911
	0–0.06 mg	−0.387	−0.329	0.388

**Table 3:** Correlation coefficients between the chelating activity and the reducing power of ethanol tea extracts and the content of thiamine pyrophosphate and thiamine pyrophosphate.

Type of Tea Extracts	Concentration of Thiamine [mg/100 g]	Correlation Coefficient of Oxidative Stability Values and Content of Thiamine			
		Chelating Activity		Reducing Power	
		Thiamine Hydrochloride	Thiamine Pyrophosphate	Thiamine Hydrochloride	Thiamine Pyrophosphate
Extract of white tea	0.08–0.8 mg	0.729	0.815	0.731	0.864
	0.2–20 mg	−0.909	−0.925	−0.882	−0.876
	0–0.06 mg	−0.090	0.355	−0.170	0.268
Extract of green tea	0.08–0.8 mg	0.611	0.817	0.691	0.640
	0.2–20 mg	−0.889	−0.887	−0.856	−0.888
	0–0.06 mg	0.307	0.298	−0.255	−0.294
Extract of yellow tea	0.08–0.8 mg	0.603	0.742	0.699	0.470
	0.2–20 mg	−0.868	−0.882	−0.788	−0.901
	0–0.06 mg	−0.061	−0.307	−0.701	−0.035
Extract of red tea	0.08–0.8 mg	0.519	0.785	0.508	0.519
	0.2–20 mg	−0.643	−0.715	−0.620	−0.628
	0–0.06 mg	0.310	0.387	0.050	0.123
Extract of black tea	0.08–0.8 mg	0.142	−0.084	−0.147	−0.069
	0.2–20 mg	−0.490	−0.416	−0.471	−0.530
	0–0.06 mg	−0.041	0.139	−0.294	0.202
Without additionals of extract	0.08–0.8 mg	0.523	0.518	0.602	0.669
	0.2–20 mg	−0.878	−0.872	−0.973	−0.961
	0–0.06 mg	0.397	0.397	0.371	0.495

**Table 4:** Correlation coefficients between the antiradical activity of tea extracts and the content of thiamine hydrochloride and thiamine pyrophosphate.

Type of Tea Extracts	Concentration of Thiamine [mg/100 g]	Correlation Coefficient of Oxidative Stability Values and Content of Thiamine			
		Chelating Activity		Reducing Power	
		Thiamine Hydrochloride	Thiamine Pyrophosphate	Thiamine Hydrochloride	Thiamine Pyrophosphate
Extract of white tea	0.08–0.8 mg	0.741	0.785	0.764	0.805
	0.2–20 mg	−0.888	−0.930	−0.913	−0.916
	0–0.06 mg	−0.023	−0.178	0.147	−0.185
Extract of green tea	0.08–0.8 mg	0.683	0.649	0.700	0.778
	0.2–20 mg	−0.884	−0.923	−0.897	−0.882
	0–0.06 mg	0.114	0.038	0.139	−0.202
Extract of yellow tea	0.08–0.8 mg	0.542	0.563	0.586	0.604
	0.2–20 mg	−0.869	−0.898	−0.836	−0.878
	0–0.06 mg	−0.061	−0.096	−0.046	−0.035
Extract of red tea	0.08–0.8 mg	0.752	0.637	0.688	0.565
	0.2–20 mg	−0.594	−0.588	−0.656	−0.613
	0–0.06 mg	0.108	0.001	−0.023	−0.006
Extract of black tea	0.08–0.8 mg	−0.220	0.764	−0.114	0.203
	0.2–20 mg	−0.586	−0.434	−0.446	−0.732
	0–0.06 mg	0.121	0.139	0.147	0.139
Without additionals of extract	0.08–0.8 mg	0,589	0,589	0,578	0,869
	0.2–20 mg	−0,985	−0,984	−0,985	−0,989
	0–0.06 mg	0,397	0,397	0,139	0,004

## Supplementary Materials

The following are available online at https://www.mdpi.com/2076-3921/8/11/555/s1, Table S1: Peroxide value to samples with thiamine hydrochloride, Table S2: Peroxide value to samples with thiamine pyrophosphate, Table S3: Anisidine value LAN to samples with thiamine hydrochloride, Table S4: Anisidine value LAN to samples with thiamine pyrophosphate, Table S5: Reducing power to samples with thiamine hydrochloride, Table S6: Reducing power to samples with thiamine pyrophosphate, Table S7: Chelating activity to samples with thiamine hydrochloride, Table S8: Chelating activity to samples with thiamine pyrophosphate, Table S9: DPPH to samples with thiamine hydrochloride, Table S10: DPPH to samples with thiamine pyrophosphate, Table S11: ABTS to samples with thiamine hydrochloride, Table S12: ABTS to samples with thiamine pyrophosphate.
