# Supplementary material for: The Effect of Thiamine Concentration on the Antioxidative Activity Indices in Tea Extracts
Source: Antioxidants (Basel). 2019 Nov 15;8(11):555. doi: 10.3390/antiox8110555 (PMC6912681; doi:10.3390/antiox8110555)
Supplement: Supplementary file 1 [file antioxidants-08-00555-s001.pdf]

antioxidants MDPI

Table S1. Peroxide value to samples with thiamine hydrochloride.

Thiamine (mg/100g)	Peroxide Value Oil without Additionals Peroxide Value	Oil + White Tea Peroxide Value	Oil + Green Tea Peroxide Value	Oil + Yellow Tea Peroxide Value	Oil + Red Tea Peroxide Value	Oil + Black Tea Peroxide Value
0mg	21.75	14.96	15.66	15.97	16.25	16.74
0.01mg	21.78	14.99	15.68	15.96	16.28	16.77
0.02mg	21.15	15.24	15.76	15.94	16.15	16.76
0.04mg	21.36	15.17	15.64	15.99	16.11	16.74
0.06mg	21.62	14.99	15.71	15.95	16.28	16.75
0.08mg	21.15	14.94	15.65	15.97	16.14	16.77
0.1mg	20.81	14.55	14.94	15.98	16.23	16.73
0.2mg	20.79	14.25	14.92	15.79	16.13	16.76
0.4mg	20.77	13.88	14.77	15.78	16.10	16.73
0.8mg	20.71	13.34	14.75	15.78	16.11	16.75
1mg	21.78	14.46	16.30	15.77	15.79	16.14
1.4mg	21.81	15.95	16.77	16.09	16.24	16.42
2mg	21.84	16.82	17.13	16.77	17.13	16.35
3mg	22.67	17.81	17.53	16.88	17.68	16.46
4mg	22.77	17.95	17.61	18.42	15.88	16.37
6mg	22.86	18.10	17.82	18.77	16.68	16.36
8mg	23.69	18.22	17.65	18.77	17.88	16.18
9mg	24.18	18.54	18.18	18.66	17.57	16.90
13.5mg	25.68	20.03	19.70	18.68	19.14	16.83
16mg	26.27	21.57	20.33	19.77	18.81	16.66
18mg	26.41	21.88	20.88	19.90	19.68	17.40
20mg	27.70	23.77	20.88	19.78	17.88	17.54

Table S2. Peroxide value to samples with thiamine pyrophosphate.

Thiamine (mg/100g)	Peroxide Value Oil without Additionals Peroxide Value	Oil + White Tea Peroxide Value	Oil + Green Tea Peroxide Value	Oil + Yellow Tea Peroxide Value	Oil + Red Tea Peroxide Value	Oil + Black Tea Peroxide Value
0mg	14.96	10.17	10.87	11.18	11.46	11.95
0.01mg	14.09	10.20	10.89	11.18	11.39	11.96
0.02mg	14.96	10.25	10.77	11.19	11.42	11.98
0.04mg	14.92	10.24	10.65	11.15	11.39	11.95
0.06mg	14.03	10.16	10.98	11.19	11.45	11.95
0.08mg	14.98	10.21	10.30	10.50	11.45	11.96
0.1mg	13.72	10.00	10.10	10.38	11.39	11.17
0.2mg	12.70	9.75	9.90	10.10	11.20	11.89
0.4mg	13.23	9.50	9.75	10.00	11.20	11.09
0.8mg	13.32	9.34	9.25	9.79	10.67	11.03
1mg	14.54	9.87	9.66	10.00	11.05	11.67
1.4mg	14.99	10.00	11.35	11.45	11.45	10.52
2mg	14.96	11.20	11.52	12.00	11.67	11.98
3mg	15.02	11.34	11.89	12.12	12.99	10.49
4mg	15.12	11.67	11.60	11.63	12.45	11.89
6mg	15.91	12.00	11.81	11.84	13.65	10.56
8mg	15.74	12.30	11.99	11.67	11.79	12.45
9mg	16.23	12.78	13.00	13.00	11.98	11.51
13.5mg	17.73	13.42	14.56	13.70	13.82	11.99
16mg	18.32	14.96	15.21	13.90	13.99	12.09

18mg	18.46	16.06	15.97	14.50	14.39	13.27
20mg	18.75	17.05	16.14	15.17	15.76	13.56

Table S3. Anisidine value LAN to samples with thiamine hydrochloride.

Thiamine (mg/100g)	Anisidine Value Lan<br>Oil without Additionals Peroxide Value	Oil + White Tea Peroxide Value	Oil + Green Tea Peroxide Value	Oil + Yellow Tea Peroxide Value	Oil + Red Tea Peroxide Value	Oil + Black Tea Peroxide Value
0mg	22.08	10.37	10.87	10.89	11.34	11.55
0.01mg	22.35	10.20	10.89	10.92	11.37	11.58
0.02mg	22.09	10.20	10.77	10.79	11.24	11.45
0.04mg	22.04	10.14	10.65	10.75	11.20	11.49
0.06mg	22.10	10.32	10.98	10.92	11.37	11.58
0.08mg	22.05	10.20	10.78	10.81	11.26	11.57
0.1mg	22.01	9.86	10.55	10.58	11.23	11.54
0.2mg	21.45	9.60	10.43	10.45	11.34	11.51
0.4mg	24.24	9.50	10.43	10.43	11.08	11.56
0.8mg	24.17	9.34	10.39	10.20	11.98	11.55
1mg	25.58	10.79	12.05	14.53	14.65	12.34
1.4mg	24.36	11.98	12.68	14.64	14.76	11.49
2mg	24.42	12.42	12.74	14.71	14.83	12.02
3mg	24.72	12.42	12.74	14.71	14.83	11.79
4mg	24.56	12.56	12.82	14.79	14.91	12.20
6mg	24.71	12.71	13.03	15.00	15.12	12.23
8mg	24.61	12.61	12.86	14.83	14.97	12.25
9mg	25.20	13.16	13.39	15.36	15.48	12.28
13.5mg	26.64	14.64	14.90	16.86	15.98	12.54
16mg	28.18	16.18	16.43	16.99	14.54	12.08
18mg	29.28	17.28	17.09	17.09	16.13	12.13
20mg	29.18	17.18	17.34	17.44	16.18	12.34

Table S4. Anisidine value LAN to samples with thiamine pyrophosphate.

Thiamine (mg/100g)	Anisidine Value Lan<br>Oil without Additionals Peroxide Value	Oil + White Tea Peroxide Value	Oil + Green Tea Peroxide Value	Oil + Yellow Tea Peroxide Value	Oil + Red Tea Peroxide Value	Oil + Black Tea Peroxide Value
0mg	22.08	10.37	10.87	10.89	11.34	11.54
0.01mg	22.35	10.20	10.89	10.92	11.35	11.59
0.02mg	22.39	10.20	10.77	10.79	10.91	11.55
0.04mg	22.14	10.39	10.65	10.75	11.07	11.41
0.06mg	22.10	10.20	10.98	10.92	11.34	11.58
0.08mg	22.15	10.20	10.78	10.80	11.35	11.29
0.1mg	20.29	9.90	10.35	10.50	11.20	11.34
0.2mg	20.37	9.45	10.15	10.20	11.00	11.06
0.4mg	20.08	9.31	10.12	10.12	11.00	11.56
0.8mg	21.03	9.25	9.89	10.05	10.98	11.33
1mg	20.42	9.44	9.90	10.21	11.09	11.54
1.4mg	23.14	11.00	11.10	11.48	11.60	10.60
2mg	23.20	11.20	11.45	11.55	11.67	10.71
3mg	23.50	11.20	11.52	11.55	11.90	10.78
4mg	23.34	11.34	11.60	11.63	11.00	10.78
6mg	23.49	11.49	11.81	11.84	11.06	11.06

8mg	23.39	11.90	11.90	11.67	11.79	11.07
9mg	23.94	12.10	12.17	12.20	12.32	10.90
13.5mg	25.42	13.42	13.67	13.70	13.82	11.39
16mg	26.96	14.26	15.21	15.06	15.38	12.20
18mg	28.06	14.89	15.87	15.70	15.97	12.90
20mg	27.96	14.99	15.54	15.66	15.45	13.14

Table S5. Reducing power to samples with thiamine hydrochloride.

Thiamine (mg/100g)	Reducing Power					
	Oil without Additionals Peroxide Value	Oil + White Tea Peroxide Value	Oil + Green Tea Peroxide Value	Oil + Yellow Tea Peroxide Value	Oil + Red Tea Peroxide Value	Oil + Black Tea Peroxide Value
0mg	100.00	100.00	100.00	100.00	100.00	100.00
0.01mg	99.09	99.50	100.00	100.00	99.00	100.00
0.02mg	100.00	100.00	99.50	100.25	99.50	100.50
0.04mg	100.00	100.25	100.00	100.00	100.00	99.50
0.06mg	100.00	99.50	99.75	99.50	99.50	100.00
0.08mg	100.00	100.00	99.75	100.25	100.25	100.00
0.1mg	104.00	104.00	103.97	104.73	100.99	99.25
0.2mg	104.95	105.95	105.95	104.50	102.25	100.75
0.4mg	105.35	106.35	106.00	105.73	101.73	99.25
0.8mg	105.43	107.37	106.95	106.50	101.79	99.75
1mg	100.02	99.75	99.33	100.95	99.43	98.25
1.4mg	99.57	98.95	98.90	97.65	100.53	100.31
2mg	88.95	99.25	98.97	99.07	99.07	101.75
3mg	80.45	98.50	98.13	98.23	99.75	99.75
4mg	75.67	98.20	98.39	98.67	98.49	100.75
6mg	69.87	97.42	97.75	97.75	98.75	99.75
8mg	70.45	97.09	98.50	98.29	98.29	99.25
9mg	60.01	96.74	98.71	99.25	98.51	100.25
13.5mg	56.76	94.25	95.75	96.09	97.25	99.52
16mg	45.56	94.25	94.75	96.50	98.20	99.00
18mg	3456	90.75	91.20	95.80	99.42	99.09
20mg	27.70	89.00	89.72	91.25	98.75	98.50

Table S6. Reducing power to samples with thiamine pyrophosphate.

Thiamine (mg/100g)	Reducing Power					
	Oil without Additionals Peroxide Value	Oil + White Tea Peroxide Value	Oil + Green Tea Peroxide Value	Oil + Yellow Tea Peroxide Value	Oil + Red Tea Peroxide Value	Oil + Black Tea Peroxide Value
0mg	100.00	100.00	100.00	100.00	100.00	100.00
0.01mg	99.94	100.25	100.08	100.00	99.70	100.00
0.02mg	100.99	99.50	99.75	99.00	100.00	99.00
0.04mg	99.90	99.95	99.50	100.00	99.78	100.25
0.06mg	100.99	100.29	100.00	99.78	100.00	100.00
0.08mg	100.99	100.09	100.09	100.00	100.00	99.85
0.1mg	104.01	104.64	103.60	104.67	100.93	101.67
0.2mg	105.94	105.13	104.64	104.44	102.19	99.22
0.4mg	106.34	106.50	105.04	105.67	101.60	100.75
0.8mg	106.42	109.50	105.25	104.76	101.76	100.20
1mg	100.01	99.69	99.85	100.75	99.37	98.19
1.4mg	95.44	98.79	98.76	97.59	100.74	100.25

2mg	94.66	99.19	98.91	99.01	99.01	101.00
3mg	88.45	98.44	98.97	98.17	99.75	99.69
4mg	77.45	98.01	98.33	98.61	98.43	100.69
6mg	70.43	97.36	97.69	97.99	98.69	99.75
8mg	65.33	97.03	98.25	97.99	98.23	99.19
9mg	59.76	96.68	97.25	97.34	98.45	99.29
13.5mg	55.45	94.19	95.89	95.48	97.25	99.45
16mg	45.63	92.19	93.99	93.94	98.14	99.04
18mg	33.78	88.69	91.00	91.00	99.26	99.00
20mg	28.69	87.03	89.00	90.34	98.75	98.55

Table S7. Chelating activity to samples with thiamine hydrochloride.

	Chelating activity					
**Thiamine (mg/100g)**	**Oil without Additionals Peroxide Value**	**Oil + White Tea Peroxide Value**	**Oil + Green Tea Peroxide Value**	**Oil + Yellow Tea Peroxide Value**	**Oil + Red Tea Peroxide Value**	**Oil + Black Tea Peroxide Value**
0mg	100.00	100.00	100.00	100.00	100.00	100.00
0.01mg	99.09	100.25	99.50	100.00	99.50	100.00
0.02mg	99.89	99.50	99.98	100.89	100.00	99.78
0.04mg	100.00	100.00	100.00	100.00	99.89	100.00
0.06mg	100.00	99.96	99.95	100.09	100.00	99.95
0.08mg	100.00	100.00	100.00	100.00	99.98	100.50
0.1mg	105.85	104.50	104.98	104.58	101.24	101.98
0.2mg	104.84	105.59	105.59	105.39	102.50	99.53
0.4mg	105.98	106.55	105.98	105.98	101.98	100.50
0.8mg	106.08	107.45	106.50	106.07	102.07	101.25
1mg	89.66	105.99	104.00	104.45	101.45	100.00
1.4mg	70.66	99.91	99.80	98.43	100.78	100.56
2mg	70.98	99.34	99.22	99.32	99.32	101.40
3mg	69.33	99.24	98.38	98.48	98.48	100.98
4mg	55.78	98.54	98.50	98.92	98.74	99.50
6mg	45.45	97.67	98.00	98.65	98.68	100.50
8mg	46.43	98.34	98.44	98.54	98.54	100.75
9mg	40.33	97.99	98.06	98.36	98.76	99.87
13.5mg	39.33	95.60	96.45	97.34	97.87	100.20
16mg	26.27	94.12	95.50	95.34	98.45	99.67
18mg	26.41	90.60	91.60	92.98	99.67	100.25
20mg	27.70	88.00	89.63	90.22	98.34	98.95

Table S8. Chelating activity to samples with thiamine pyrophosphate.

	Chelating activity					
**Thiamine (mg/100g)**	**Oil without Additionals Peroxide Value**	**Oil + White Tea Peroxide Value**	**Oil + Green Tea Peroxide Value**	**Oil + Yellow Tea Peroxide Value**	**Oil + Red Tea Peroxide Value**	**Oil + Black Tea Peroxide Value**
0mg	100.34	100.00	100.00	100.00	100.00	100.00
0.01mg	99.43	99.89	100.08	100.00	99.85	100.00
0.02mg	100.23	100.00	99.50	100.89	100.00	99.23
0.04mg	100.34	100.98	100.98	100.00	100.56	100.00
0.06mg	100.34	100.00	100.00	99.78	100.00	100.00
0.08mg	100.34	100.09	100.09	100.00	100.00	100.00
0.1mg	106.19	103.89	104.99	104.95	103.20	100.23
0.2mg	105.18	105.99	105.39	105.35	102.34	99.89

0.4mg	105.43	107.98	107.99	107.50	104.00	100.25
0.8mg	106.42	108.95	109.35	108.00	104.95	100.00
1mg	89.00	101.00	102.90	100.00	99.68	99.68
1.4mg	70.01	99.90	100.00	99.90	100.78	100.56
2mg	70.33	99.34	99.22	99.75	99.95	99.32
3mg	68.68	99.24	98.38	99.00	98.48	98.48
4mg	55.13	98.90	98.64	98.92	98.74	98.74
6mg	44.80	98.35	98.55	98.65	98.99	100.23
8mg	45.78	97.45	98.44	98.54	98.54	100.34
9mg	39.68	96.80	98.66	97.90	98.76	99.87
13.5mg	38.68	93.50	94.42	96.34	97.87	97.50
16mg	28.98	90.56	93.45	94.32	98.45	99.67
18mg	26.77	88.05	90.66	90.56	97.89	99.34
20mg	27.05	87.05	89.42	89.52	96.89	98.65

Table S9. DPPH to samples with thiamine hydrochloride.

Thiamine (mg/100g)	reducing power					
	Oil without Additionals Peroxide Value	Oil + White Tea Peroxide Value	Oil + Green Tea Peroxide Value	Oil + Yellow Tea Peroxide Value	Oil + Red Tea Peroxide Value	Oil + Black Tea Peroxide Value
0mg	100.00	100.00	100.00	100.00	100.00	100.00
0.01mg	99.09	99.50	99.50	100.00	100.15	100.25
0.02mg	99.89	100.00	99.98	100.89	100.00	99.78
0.04mg	100.00	100.00	100.25	100.00	99.89	99.78
0.06mg	100.00	99.75	99.75	100.09	100.15	100.25
0.08mg	100.00	100.00	100.00	100.00	99.98	100.50
0.1mg	105.98	104.08	104.98	104.98	101.24	101.98
0.2mg	104.89	105.99	104.99	104.99	102.50	99.53
0.4mg	105.98	106.45	105.98	105.98	101.98	100.50
0.8mg	106.76	107.58	106.89	105.67	102.98	100.45
1mg	101.09	99.88	101.20	100.30	99.68	100.00
1.4mg	99.78	99.10	99.15	98.43	100.78	100.56
2mg	100.34	99.04	99.22	99.32	99.32	101.40
3mg	91.44	98.94	98.38	98.48	98.48	100.98
4mg	89.55	98.04	98.64	98.92	98.74	99.50
6mg	71.24	97.67	98.00	98.65	98.68	100.50
8mg	68.97	97.34	98.44	98.54	98.54	100.75
9mg	61.35	96.21	98.66	98.76	98.76	99.87
13.5mg	56.33	94.50	95.45	96.34	97.87	100.20
16mg	46.24	94.12	95.50	95.74	98.45	99.67
18mg	40.24	90.60	90.66	91.98	99.67	99.34
20mg	28.60	88.00	89.03	89.52	98.34	98.90

Table S10. DPPH to samples with thiamine pyrophosphate.

Thiamine (mg/100g)	reducing power					
	Oil without Additionals Peroxide Value	Oil + White Tea Peroxide Value	Oil + Green Tea Peroxide Value	Oil + Yellow Tea Peroxide Value	Oil + Red Tea Peroxide Value	Oil + Black Tea Peroxide Value
0mg	100.00	100.00	100.00	100.00	100.00	100.00
0.01mg	99.09	99.89	99.50	99.50	99.85	100.00
0.02mg	99.89	100.40	100.00	100.89	100.00	99.23
0.04mg	100.00	99.75	99.50	100.00	100.56	100.00

0.06mg	100.00	100.00	100.00	99.78	99.70	100.00
0.08mg	100.00	100.09	100.09	100.00	100.00	100.00
0.1mg	105.98	104.98	103.99	104.90	101.23	100.23
0.2mg	104.89	103.99	104.39	104.99	102.34	99.89
0.4mg	105.98	106.98	104.99	105.98	102.00	100.25
0.8mg	106.76	107.95	105.43	105.77	102.32	100.45
1mg	100.98	105.40	102.58	102.30	99.68	99.68
1.4mg	99.89	104.80	99.15	98.43	100.78	100.56
2mg	99.78	99.34	99.22	99.32	99.32	99.32
3mg	90.54	99.24	98.38	98.48	98.48	98.48
4mg	89.22	98.54	98.64	98.92	98.74	98.74
6mg	70.34	97.45	98.55	98.65	98.68	100.23
8mg	68.43	98.34	98.44	98.54	98.54	100.34
9mg	60.45	98.56	98.66	98.76	98.76	99.87
13.5mg	55.43	94.32	94.42	96.34	97.87	97.56
16mg	45.34	93.12	94.22	94.32	98.45	99.67
18mg	39.34	90.56	90.66	88.98	99.67	99.34
20mg	27.70	87.45	89.42	89.52	98.56	98.67

Table S11. ABTS to samples with thiamine hydrochloride.

**Thiamine (mg/100g)**	**reducing power**					
	**Oil without Additionals Peroxide Value**	**Oil + White Tea Peroxide Value**	**Oil + Green Tea Peroxide Value**	**Oil + Yellow Tea Peroxide Value**	**Oil + Red Tea Peroxide Value**	**Oil + Black Tea Peroxide Value**
0mg	100.00	100.00	100.00	100.00	100.00	100.00
0.01mg	100.00	99.25	100.00	100.00	99.00	99.25
0.02mg	99.50	100.00	99.50	100.75	100.00	100.00
0.04mg	100.00	99.50	100.00	100.00	100.00	99.50
0.06mg	100.00	100.00	100.00	100.09	99.50	100.00
0.08mg	100.00	100.00	100.00	100.00	100.50	100.00
0.1mg	104.50	104.23	103.98	104.98	101.24	99.50
0.2mg	105.70	105.70	105.77	104.75	102.50	101.00
0.4mg	105.98	106.45	105.55	105.98	101.98	99.50
0.8mg	105.98	107.77	106.81	105.91	102.54	99.93
1mg	99.98	103.09	102.99	101.20	101.08	98.50
1.4mg	99.89	99.99	99.15	97.90	100.78	100.56
2mg	97.34	99.50	99.22	99.32	99.32	102.00
3mg	90.54	98.75	98.38	98.48	100.00	100.00
4mg	87.45	98.00	98.64	98.92	98.74	101.00
6mg	70.34	97.45	98.00	98.30	99.00	100.00
8mg	68.43	96.05	98.75	98.54	98.54	99.50
9mg	61.34	95.64	98.66	99.50	98.76	100.50
13.5mg	44.78	94.89	94.00	96.34	97.50	99.77
16mg	45.20	93.45	94.22	96.75	98.45	99.32
18mg	39.12	90.00	92.45	93.45	99.67	99.34
20mg	27.70	88.00	89.45	90.45	99.00	98.89

Table S12. ABTS to samples with thiamine pyrophosphate.

**Thiamine (mg/100g)**	**reducing power**					
	**Oil without Additionals Peroxide Value**	**Oil + White Tea Peroxide Value**	**Oil + Green Tea Peroxide Value**	**Oil + Yellow Tea Peroxide Value**	**Oil + Red Tea Peroxide Value**	**Oil + Black Tea Peroxide Value**

0mg	100.00	100.00	100.00	100.00	100.00	100.00
0.01mg	100.00	99.89	100.15	100.00	100.50	100.00
0.02mg	101.22	99.75	99.75	99.00	100.00	99.00
0.04mg	99.98	99.50	99.85	100.00	100.56	100.00
0.06mg	100.23	100.00	100.00	99.78	100.00	100.00
0.08mg	101.23	100.09	100.09	100.00	100.00	100.00
0.1mg	103.56	104.50	103.92	104.92	101.18	101.92
0.2mg	102.33	105.50	104.81	104.69	102.44	99.47
0.4mg	105.23	106.98	105.49	105.92	102.92	101.00
0.8mg	106.23	108.50	106.94	105.99	102.24	101.04
1mg	105.23	103.45	99.52	101.14	99.62	101.23
1.4mg	99.89	100.25	99.09	99.95	100.72	100.50
2mg	98.98	99.44	99.16	99.26	99.26	101.94
3mg	92.34	98.69	98.32	98.95	99.94	99.94
4mg	89.45	98.31	98.58	98.86	98.68	100.94
6mg	75.56	97.96	98.24	98.94	98.94	100.00
8mg	67.45	97.23	98.50	98.98	98.48	99.44
9mg	63.24	96.70	97.50	99.44	98.70	99.50
13.5mg	50.98	94.50	93.94	96.28	97.50	99.71
16mg	45.30	93.50	94.16	95.10	98.39	99.26
18mg	40.23	90.00	91.39	93.45	99.61	99.28
20mg	30.22	87.05	90.97	92.75	98.94	98.95
